# Influence of Personality Traits and Its Interaction with the Phenomenon of Bullying: Multi-Centre Descriptive Study

**DOI:** 10.3390/ijerph17010172

**Published:** 2019-12-25

**Authors:** Manuel Pabón-Carrasco, Lucia Ramirez-Baena, Nerea Jiménez-Picón, José Antonio Ponce Blandón, José Manuel Martínez-Montilla, Raúl Martos-García

**Affiliations:** Spanish Red Cross Nursing School, Universidad de Sevilla, Avda. de la Cruz Roja, nº 1 Dpdo., 41009 Seville, Spain; mpabon@cruzroja.es (M.P.-C.); nejipi@cruzroja.es (N.J.-P.); japonce@cruzroja.es (J.A.P.B.); josmarmon3@alum.us.es (J.M.M.-M.); rmartos@cruzroja.es (R.M.-G.)

**Keywords:** adolescence, aggression, bullying, nursing, personality, schools

## Abstract

Bullying affects thousands of teenagers worldwide and has devastating consequences. Various studies suggest that the personality of teenagers is a risk profile for bullying. The aim of this study was to analyse the relationship between the personality of teenagers aged 14 to 16 years from three education centres located in the province of Seville (Spain) and bullying in any of its victim or aggressor roles. A multi-centre cross-sectional observational descriptive study was conducted in three education centres in the province of Seville (Spain). The sample consisted of 93 students. In order to measure the two main variables, the Bull-S test was used for bullying, and the EPQ-J questionnaire was used for personality traits. A descriptive and correlation analysis was performed between variables. The results showed that 14% (*n* = 13) of the sample were detected as victims and another 14% (*n* = 13) were detected as aggressors. Statistically significant differences were found between neuroticism (*p* = 0.044; Phi = 0.615), sincerity (*p* = 0.016; V de Cramer = 0.474), and anti-social behaviour (*p* = 0.007; Phi = 0.620) with the variables victim/aggressor. Bullies are typically males who score high on neuroticism and anti-social behaviour, with a tendency towards social dissimulation.

## 1. Introduction

The phenomenon of bullying has been increasingly researched in recent years because of the increase in the number of cases worldwide—two out of ten teenagers claim to have suffered bullying at least once in their life [1]. This serious public health issue affects teenagers all over the world. It indirectly causes at least 200 deaths every year [2]. Though social awareness is growing, recent years have seen a greater number of cases diagnosed [3].

### 1.1. The Phenomenon of Bullying

This term, proposed by Olweus in the 1970s, refers to a specific form of continued peer-to-peer school violence in which one or more aggressors have greater power and intentionality to cause pain with violence subjected to a school/institution partner (victim) who is weaker [2].

In the phenomenon of bullying, there are two main roles—the victim (the person who is subdued and abused) and the aggressor (the person who subdues the victim by force)—and one additional role, which is increasingly frequent but still critical: the spectator (the person who watches attacks and even films them without mediating the situation) [4]. Victims are usually students with social disadvantages compared to other peers, with a tendency to social isolation, and are introverted and vulnerable due to some physical, psychological, or academic peculiarity [5]. Aggressors are often in a position of power with respect to victims; they are older, extroverted, prone to misconduct, and usually acting as a group [5].

The phenomenon characteristics, such as the persistence, time, severity, and power relationships, are variables that influence the biopsychosocial impact in the short-, medium-, and long-term [6]. The stages of bullying are progressive, gradually increasing in severity [7,8,9].

Bullying comprises all types of violence: verbal, physical, social (social exclusion, margination, etc.), psychological, and even violence through new technologies and social media (cyberbullying or “happy slapping”) [10,11,12]. All these new forms of bullying have led to the updating and criminalisation of offences internationally in order to address this phenomenon. In fact, in 2015, Spain reformed the Criminal Code to introduce a new offence against freedom known as “stalking” [13].

The consequences of bullying are serious and can even extend to the victim’s adulthood [14]. The longer it takes to intervene, the more serious the consequences will be [15]. The victim enters a cycle of sadness, anxiety, and fear that persists for a long time period, even after the bullying itself has ended [16]. This can lead them to commit self-harm and suicide [6,7,8,9,17,18].

Suicide is the third cause of death in the 15 to 29 age group in Spain [19]. Finland, England, Russia, and Spain are among the countries with higher suicide rates [1,2]. The World Health Organisation (WHO) recognises suicide as a public health priority. 

### 1.2. Bullying in Adolescence and its Influence on Personality

Adolescence is a stage characterised by personality changes and a greater vulnerability to suicide when social groups have a decisive influence on the affective and social world of teenagers [20]. Additionally, personality, identity, social recognition, and emotional stability [21,22] are formed during this stage, and consequently, teenagers are a vulnerable group to bullying.

Even though personality is not fully defined during adolescence, several studies have explored the relationship between personality and the victim, aggressor, and spectator roles [23,24,25]. Nevertheless, it is still unclear whether it is a risk factor or a protective factor, unlike other constructs that imply a risk factor with respect to bullying, such as narcissism [26]. In contrast, empathy [27,28] and the ability to forgive [29] have been found to be a protective factor against this phenomenon.

Prevention is critical to prevent the phenomenon of bullying and its terrible consequences from ending the victim’s life [30]. There are several people and professionals involved in the prevention of bullying who can act on all three levels of attention, from parents, teachers, and counsellors to health workers and even the media [31]. Europe is introducing the figure of the school nurse, who may be a critical identifier of potential cases of bullying and whose role is fundamental in preventing this phenomenon [32]. 

If the characteristic personality traits of teenagers are unknown, in order to analyse if they are linked to a victim or aggressor profile within the roles of bullying, this figure will facilitate the prevention and diagnose of such phenomenon in education centres. As bullying is a major challenge, it is imperative to globally introduce the figure of a school nurse who, along with the counselling and psychology team of the school or high school, can prevent, detect, and tackle this kind of situations on time.

So the hypotheses formulated to conduct this investigation are: H_1_ = there is no predictive correlation between Bull-S test variables and personality traits according to the EPQ-J questionnaire; H_2_ = aggressors have a risk profile prone to neuroticism, extraversion, and a greater tendency to social dissimulation; H_3_ = victims have a risk profile prone to psychoticism, introversion, and with a tendency to sincerity; H_4_ = both roles (victim/aggressor) have anti-social behaviour. 

Therefore, the objective of this study is to analyse the relationship between the personality of teenagers aged 14 to 16 years from three education centres located in the province of Seville (Spain) and the bullying in any of its victim or aggressor roles.

## 2. Method

### 2.1. Design

A multi-centre cross-sectional observational descriptive study was conducted in three education centres in the province of Seville (Spain).

#### 2.1.1. Sample/Participants

The sample consisted of 93 students recruited through convenience sampling between the ages of 14 and 16 and in their third year of secondary education during the school year 2018/2019. The sample was taken from three different centres in the province of Seville (Spain): two State-subsidised private schools and one public school. Out of an initial selection of 102 participants, the sample was finally reduced to 93 students with a response rate of 89.4%. 

The sample size was calculated for a power of 0.95, with an alpha error of 0.05 and with medium size effect (G*Power 3.1.9.4, Franz Faul, Kiel University, Kiel, Germany). A total sample of 111 participants was estimated to be necessary.

#### 2.1.2. Recruitment Process

Participation was anonymous and voluntary, and students had the right to drop out at any time. Firstly, the heads of the education centres were contacted by phone and invited to take part in the study with their students. The counsellors, along with the Management Board, approved the development of the study in the different centres. Secondly, parents/guardians were given the informed consents, which had to be necessarily signed by them for students to participate in the study. The exclusion criterion was the age (14–16 years old), excluding only repeaters who exceeded the predetermined age limit, students from other years, and students who were absent during the procedure.

Investigators distributed the questionnaires among students during tutoring class with the collaboration of teachers, and counsellors monitored the filling of such questionnaires in order to guarantee privacy and avoid conditioning the answers of the students at all times. Out of an initial selection of 102 participants, the sample was finally reduced to 93 students due to the lack of answers in the questionnaires used.

### 2.2. Data Collection

The study variables were socio-demographic and were collected by means of an ad hoc questionnaire on the age, sex, education centre, and school year of students, as well as on whether they had repeated a year so as to identify repeaters as a possible moderating variable. 

The variables included in the study were coding as follow: student profile: 1 = aggressor, 2 = victim; type of abuse: 0 = no, 1 = insulting and threatening, 2 = physical abuse, 3 = rejection, 4 = other; place where abuse occurs: 0 = no, 1 = in the classroom, 2 = at the playground, 3 = in the corridors, 4 = other places; frequency of abuse: 0 = every day, 1 = once/twice a week, 2 = rarely, 3 = never; perception of severity: 0 = hardly or non-severe, 1 = somewhat severe, 2 = quite severe, 3 = very severe; perception of safety in the education centre: 0 = unsafe or hardly safe, 1 = somewhat safe, 2 = quite safe, 3 = very safe.

In order to measure the two main variables of the study, the Bull-S test was used for bullying, and the EPQ-J questionnaire was used for personality traits.

#### 2.2.1. Bull-S Test 

Based on the methodological approach of sociometry, the study of group development and the position of individuals within groups [33], the Bull-S test is a collectively applicable self-administered questionnaire specifically elaborated to measure peer aggressiveness in school settings [34]. It is aimed at a child/adolescent population aged between 7 and 16 years, and it takes around 25 to 30 min to complete it.

It consists of 15 items and addresses three dimensions: the study of the internal classroom structure; the aggressiveness-victimisation, including nominally; and situational variables, like the type of abuse, the place where the bullying occurs, the frequency of abuse, the assessment of the situation, and the safety perceived in the education centre [35]. The following are examples of the items that are evaluated in the test: “Who would you choose as a classmate in class?” and “Who usually starts the fights?”.

This questionnaire is validated in the Spanish population and has adequate internal validity and reliability with a Cronbach’s alpha of 0.69 to 0.75 [36].

#### 2.2.2. EPQ-J Questionnaire 

Based on Eysenck’s multifactorial model of personality [37], it is a self-administered questionnaire composed of 81 items with dichotomous responses (Yes/No), takes around 20 min, and is aimed at the child/adolescent population aged between 7 and 16 years [38].

It assesses three basic dimensions of personality plus one additional dimension focused on sincerity: neuroticism (N), with 20 items; extraversion (E), with 24 items; psychoticism (P), with 17 items; and sincerity (S), with 20 items. Moreover, this questionnaire has an additional scale assessing anti-social behaviour (AB), composed of 40 items included in dimensions N, E, and P, which aims to predict criminal behaviour [39]. The following are examples of the items that are evaluated in the test: “Are you very happy and lively?” and “Do you often like being alone?”.

This questionnaire is validated in the Spanish population [38] and has adequate internal validity and reliability for each of the three dimensions: Cronbach’s alpha of 0.78 for N, 0.73 for E, 0.68 for P, and 0.77 for S [40]. The additional anti-social behaviour scale has a Cronbach’s alpha of 0.87 [41]. 

### 2.3. Ethical Considerations

The data were processed in accordance with the provisions of Act 3/2018, of 5 December, on the Protection of Personal Data, and the ethical considerations of the Declaration of Helsinki were complied with at all times. 

All the participating students submitted the informed consent signed by their parents/guardians and the consent and supervision of the heads of each education centre. The Research Commission of the Nursing University Centre Cruz Roja in Seville (21102019CUECR01) granted its approval.

### 2.4. Data Analysis 

A descriptive analysis of the data was conducted, calculating measures of central tendency (mean and standard deviation) for quantitative variables, and the absolute and relative frequency for qualitative variables. 

A chi-square test was used to compare qualitative variables, setting the statistical significance level at *p* < 0.05. In the case of statistical significance, its magnitude was studied determining Cramer’s V or phi coefficient depending on whether there was a number of different categories between variables, using binary or multinomial logistic regression models, where associations were estimated through the odds ratio (OR).

In the case of an analysis of association between qualitative and quantitative variables, the Kolmogorov–Smirnov normality test was performed in order to determine parametric or non-parametric tests. Kruskal–Wallis/Mann–Whitney *U* test was used for non-parametric tests depending on the number of categories of the qualitative variable, and Student *t*-test/ANOVA test was used for parametric tests. In case of statistical significance, its magnitude was studied using Hedges’ *g* index for factors with two possible values that resulted significant, in the case of the Mann-Whitney *U* test. 

Finally, in the case of an analysis of association between quantitative variables, a variate correlation between both was performed, determining Pearson’s correlation coefficient. 

Analyses were conducted with the statistical package SPSS 25.0 (IBM Corp., Armonk, NY, USA).

## 3. Results

### 3.1. Characteristics of the Study Sample

A total of 58.1% (*n* = 54) of the study population were females and 41.9% (*n* = 39) were males. The average age of participants was 14.72 years (SD 0.79; CI95%, 14.56–14.88). Of the participants, 18.3% (*n* = 17) claimed to have repeated some year vs. 81.7% (*n* = 76) who did not.

The total sample of 93 students was collected from three different centres in the province of Seville (Spain): two concerted centres (C_1_ *n* = 21, C_2_ *n* = 30) and one public (C_3_ *n* = 42). The gender distribution was mostly women in all three centres (C_1_ 76% females and 24% males; C_2_ 61% females and 39% males; C_3_ 52% females and 48% males). The average age of participants in each centre was: C_1_ 14.27 ± 0.61; C_2_ 14.06 ± 0.52; C_3_ 14.71 ± 0.81. There were two repeaters in C_1_, five in C_2_, and 10 in C_3_. The victims identified were four in C_1_, three in C_2_, and six in C_3_. The aggressors identified were four in C_1_, four in C_2_, and five in C_3_.

### 3.2. Descriptive Data on the Phenomenon of Bullying in this Study

In order to identify the profile of students through the Bull-S test, a socio-matrix was built for the first and second dimensions, identifying that 28% (*n* = 26) of the sample had victim and aggressor roles—specifically, 14% had victim roles and 14% had aggressor roles, as shown in Table 1.

Within both profiles, in terms of sex, 53.8% (*n* = 14) were females and 46.2% (*n* = 12) were males, with a majority of females in the victim profile (61.5%, *n* = 8) and a majority of males in the aggressor profile (58.3%, *n* = 7). The average age of the victims identified in the study was 14.62 years old (SD 0.87; CI95%, 14.09–15.14), while that of the bullies was 14.46 years old (SD 0.52; CI95% 14.15–14.78). Only one repeater subject was found in the victim profile (7.7%, *n* = 1), as occurred in the aggressor profile (7.7%, *n* = 1).

The responses about the third dimension of the Bull-S test are reflected in Table 1.

### 3.3. Descriptive Data on the Personality Traits of the Study Participants

The scores obtained in the different dimensions analysed in the EPQ-J questionnaire for the students who participated in this study are shown in Table 2.

The scale of anti-social behaviour was also analysed using percentiles, where 33.3% (*n* = 31) have a low tendency, while 34.4% (*n* = 32) and 32.3% (*n* = 30) have a moderate and a high tendency, respectively. In this last scale, an average score of 19.89 was obtained (SD 7.09; CI95%, 18.43–21.35).

### 3.4. Correlation Between Socio-Demographic Variables and Bullying

The determination of the possible relationship of responses in the third dimension of the Bull-S test, which analyses the perception of the frequency, the place where abuse occurs, etc., resulted in statistically significant differences between males and females with respect to the perception of the frequency of abuse (*p* = 0.028; phi = 0.317). It has been noted that males perceive abuse as “rarely” occurring, in contrast with females, who perceive it as “never” occurring (OR = 0.260). Meanwhile, no relationship has been observed between age and repeating a year with the variables of the second part of the Bull-S test, such as the type and frequency of bullying, the place where it occurs, the severity of abuse and the level of safety in the education centre.

### 3.5. Correlation Between Socio-Demographic Variables and Personality Traits 

No statistically significant correlations were identified between sex and personality variables, assessed through EPQ-J questionnaire.

As regards the correlation between age and personality variables, a statistically significant correlation was observed in the neuroticism dimension (*p* = 0.024; H of Kruskal-Wallis = 11.288). 

This is also true for the correlation of variable repeating a year with personality traits, where neuroticism stands out again as statistically significant (*p* = 0.043; phi = 0.325). The multinomial regression resulted in an OR = 4.500 among non-repeaters with an unstable emotional tendency in the neuroticism dimension, in comparison with repeaters who had a stable emotional tendency. There is also a correlation with the anti-social behaviour scale in repeaters (*p* = 0.046; phi = 0.257): OR of 3.333 and 4.286 in non-repeaters with an intermediate and high score, respectively, on the anti-social behaviour scale, with respect to a repeater with a score <16.85 on the same scale.

### 3.6. Correlation Between Bullying and Personality Variables 

Regarding the dimensions studied in the EPQ-J questionnaire, statistically significant differences have been found between neuroticism (*p* = 0.044; phi = 0.615), the sincerity dimension (*p* = 0.016; Cramer’s V = 0.474), and the anti-social behaviour scale (*p* = 0.007; phi = 0.620) with the variable victim/aggressor. Concerning the relationship between victim/aggressor profiles and the neuroticism dimension, OR = 17.658 in a student with a tendency to neuroticism and an aggressor profile, while OR = 10.634 in a non-neurotic person with a victim profile. 

With respect to the possible relationships between the various dimensions of the EPQ-J questionnaire, a correlation between P and S was observed (*p* = 0.019; phi = 0.279), as well as an OR = 17.170 among students who were highly stable and tended to sincerity and stability in the psychoticism scale, in comparison with an OR = 56.196 in unstable students with a tendency to social dissimulation. Likewise, a correlation was found between the neuroticism dimension and the additional anti-social behaviour scale (*p* = 0.001; phi = 0.529).

By determining the relationship between victim/aggressor profiles and the sincerity dimension, it was found that a student with an aggressor profile and a tendency to sincerity had an OR = 0.114, in comparison to a victim with a tendency to social dissimulation.

Regarding the relationship between victim/aggressor profiles and the anti-social behaviour scale, it was found that OR = 32.446 in students who were considered as bullies by their classmates and had a high to intermediate score in the anti-social behaviour scale, and OR = 2.684 in students described as victims who had a low score in the anti-social behaviour scale. 

Statistically significant differences were also found between the victim and aggressor profiles in the anti-social behaviour scale (*p* = 0.007; phi = 0.620), where the average score of participants described as victims was 17.46 points (SD 10.68; CI95%, 11.01–23.92), and the average score of bullies was 29.62 points (SD 2.53; CI95%, 28.08–31.15). 

The Alpha Conbrach of each test used in the study was calculated with 0.71 for Bull-S test and of 0.73 for N, 0.67 for E, 0.69 for P, 0.72 for S, and 0.80 for AB. Table 3 shows the correlations of the variables included in the EPQ-J questionnaire with each other, as well as the variables of the second part of the Bull-S test with each other.

## 4. Discussion

The objective of this study is to analyse the relationship between the personality of teenagers aged 14 to 16 years from three education centres located in the province of Seville (Spain), and bullying in any of its victim or aggressor roles. Out of the 93 students comprising the sample, 14% matched the victim profile, and 14% matched the aggressor profile. This percentage is in line with other published articles [42], though most studies in this field have found more bullies than victims [43,44]. This parity in the results of both roles may be due to the fact that the sample is comprised by students from three different centres in the province of Seville, as well as the possible lack of sincerity in the responses of students in the Bull-S test, considering that most study subjects (55.9%) tend to social dissimulation, which may have undermined the detection of possible bullies, as other authors point out [45]. 

According to the results, the pattern of a majority of females in the victim profile (61.5%) and a majority of males in the aggressor profile (58.3%) repeats itself again, as well as in most published studies about the phenomenon of bullying [46]. Nevertheless, this pattern is reversing—bullying is increasingly identified among females [47], understanding bullying as rejection and/or verbal, physical, or any other kind of abuse. 

It is worth noting that the average age of victims is higher than that of bullies, which contradicts most studies in the field [48], though the difference is minimal (14.62 years for victims and 14.46 years for bullies).

Since the percentage of repeaters is the same (7.7%) in victims and bullies, it cannot be concluded that the variable repeating a year is a moderating variable to predict the victim or aggressor profile, despite the findings of other publications [49]. However, there were statistically significant differences between repeating a year and neuroticism (*p* = 0.043), as well as anti-social behaviour (*p* = 0.046). These subjects define themselves as anxious, troubled, fearful to change, and prone to mood swings and depression [50], which may correspond to some of the feelings reported in studies conducted in repeaters. Many studies have already demonstrated a correlation between anti-social behaviour and being a repeater [51], and some authors even suggest a possible prediction of criminal behaviour [52]. 

The comparison of data by concerted centres vs. public centres, taking into account that the sample size is larger in public centres, the results show a higher percentage of victims and aggressors identified in it, although there are usually no differences in the phenomenon of bullying depending on the type of centre.

With regard to abuse analysis, the majority of the sample agreed that the most common type of abuse was rejection (44.1%), which supports the theory that rejection, indifference, social isolation, and contempt are the most frequent and unnoticed forms of bullying [53]. Therefore, both parents/guardians and professionals involved in the early detection and tackling of bullying, such as teachers, counsellors, and school nurses, must be sensitised and understand rejection as a type of abuse [54].

The same applies to the place where abuse occurs since the results of this study point to the classroom (53.8%), where the teacher is usually present, while other authors state that the playground is the most common place for bullying [55]. All this highlights the imperative need to train teachers in early detection of this phenomenon in order to avoid its unstoppable development [54,56].

With regard to the frequency of abuse, most students claimed it was rare, with a significant difference between females and males (*p* = 0.028; phi = 0.317). The latter perceived abuse as “rarely” occurring, while females perceived it as “never” occurring (OR = 0.260). There is little literature on the subject, but this difference between sexes may be due to the fact that males and females have a different understanding of the violence model [57], though such understanding is currently changing. Moreover, some authors state that aggressiveness is stronger in males and abuse is more frequent in a male environment, which makes them detect it more frequently, so rejection, margination, and exclusion are less detected and more restricted to the female environment [58]. 

The severity of such abuse is also noteworthy, considering that 42.5% of the sample describes it as hardly or non-severe. This is one of the main problems of bullying, which society has had to fight and currently fights to eradicate [59]. In the past, abuse by teachers to students, as well as among schoolmates, was justified and normalised in the education society. Today there is zero tolerance for any aggressive attitude or intimidation among schoolmates, but there remains an underlying cultural subconsciousness that cannot fully see the problem, not giving it the importance it deserves.

However, the data confirm that students feel very safe in all three education centres with 73.3% (quite safe with 33.3% (*n* = 30) and very safe with 40.0% (*n* = 36)), which contradicts the abovementioned data, liking the place where the abuse occurs (the classroom) or the perception of physical abuse in 10.8% of students, which reflects a poor safety climate in the centre, as concluded by other studies [60]. Some authors point out that a more authoritarian policy at the centre does not prevent the problem of bullying. To eradicate it and increase the feeling of security by the students in the centre, the heads of the education centres have to work the most basic levels of education into students and improve the organisation and preparation of the teachers [61].

In respect of personality variables, the neuroticism dimension showed statistically significant differences with age (*p* = 0.024), which is also pointed out by other authors [62]. Older students are more troubled, anxious to have things under control, fearful to change, and prone to depression [63]. In addition, victim and aggressor profiles had statistically significant differences with respect to neuroticism (*p* = 0.044; phi = 0.615), which was higher in bullies than victims (OR = 17.658 and 10.634). 

The same is true for sincerity (*p* = 0.016; Cramer’s V = 0.0474) since a student with an aggressor profile and a tendency to sincerity had an OR = 0.114 in comparison to a victim with a tendency to social dissimulation. All this supports the idea that bullies lie in order to cover up abuse and avoid liabilities, even criminal ones [64].

Finally, anti-social behaviour also showed statistically significant differences (*p* = 0.007; phi = 0.620) with the variable victim/aggressor. Bullies obtained a high or intermediate score on this scale, while victims had a low score in anti-social behaviour (OR = 32.446 and 2.684). In a Peruvian study, the cut-off score was 16.83 as low in anti-social behaviour, while 24.87 was high on the same scale [65]. These results are in line with the percentiles and scores obtained in this study, where the average score of victims is 17.46 points and that of bullies is 29.62 points.

These results confirm, once again, the current bullying situation, which devastates and affects not only Spanish young people but young people worldwide, taking into consideration the data collected in 2017 reporting 234 cases of bullying in the region of Andalusia. Furthermore, the helpline for early detection of bullying provided by the Ministry of Education received 25,000 calls. By provinces, Madrid (170 cases) leads this terrible ranking, followed by Las Palmas (78 victims in 2017, 16% more than in 2012), Alicante (63 cases, 37% more), and Seville (57 victims, 4% more) [66]. With almost 30% of the sample concerned by one of the two main roles of this unpleasant phenomenon, including 13 victims, and all the dramatic consequences that implies, not just in adolescence, but also in adulthood, this critical issue should be urgently addressed. Therefore, further research is needed to delve into the study and profiling of victims and bullies, so as to implement preventive measures and strategies for the early detection and tackling of this phenomenon.

### Limitations

This study is cross-sectional, and despite applying correlation statistics between variables, it does not allow to discover the causal relationship between them. However, the time factor and the influence on the development of the facts analysed may be controlled by means of longitudinal designs, so undertaking this type of study in the future is considered.

There might be other unidentified variables related to the phenomenon of bullying, which would be interesting to study in future research in order to help outline the victim and aggressor profiles. For example, type of family (well-structured/unstructured), socioeconomic level, academic performance, socialisation and acceptance by peers, anxiety, and depression. It was also not possible to conclude whether being a repeater acts as a moderating variable in the fact of being a victim or an aggressor.

Future works could replicate the study in other education centres with a representative sample of the Sevillian or Spanish population. It is, therefore, necessary to undertake comparative studies between regions that allow for the estimation of the progression of bullying.

Even though all the tests used are validated and adapted to the Spanish population, with adequate validity and reliability parameters, the information collected only through questionnaires may not reflect the real situation experienced in classrooms, and bias derived from the use of questionnaires to collect information must be assumed.

## 5. Conclusions

After analysing a sample of 93 students from three education centres in the province of Seville to explore the relationship between personality variables and bullying, 13 victims and 13 bullies were found, victims being mostly females and slightly older than bullies, who were mostly males. 

There was a repeater in each profile, and statistically significant differences were found between the variable repeating a year and neuroticism, as well as anti-social behaviour, in which correlation is also demonstrated in the victim and aggressor profiles.

With respect to the variables studied in connection with the phenomenon of bullying, rejection and social exclusion are the most frequent forms of abuse, which takes place mainly in the classroom, is described as “rarely” occurring by males and as “never” occurring by females, and is not considered as severe by participants who feel very safe in their centres.

With regard to the personality variables studied, the neuroticism dimension showed statistically significant differences according to age, and between the victim and aggressor profiles, bullies being more neurotic than victims.

The same applies to sincerity. Bullies tend to social dissimulation and anti-social behaviour, considering that they obtained a high to intermediate score on the anti-social behaviour scale, while victims obtained a low score. So, the hypotheses H_2_ and H_4_ can be confirmed but not H_1_ and H_3_.

Therefore, it could be concluded, according to the results of this research, that bullies are typically males who score high on neuroticism and anti-social behaviour, with a tendency towards social dissimulation.

### Relevance to Clinical Practice and Future Research

The practical implications of this study allow to design strategies that are more focused on and adapted to the target population, raising awareness among parents and professionals involved in the education of teenagers about the type of abuse most frequently used—rejection, which most often goes unnoticed—the place where bullying occurs, its frequency, and severity, etc.

The most frequent type of abuse in bullying is social exclusion and rejection, which is most frequent in the classroom and is considered as hardly or non-severe by students, so it is critical to raise awareness among teenagers, teachers, and the rest of people involved, such as the school nurse, in order to guarantee the prevention and early detection of this phenomenon. Additionally, these results are an important basis for developing risk profiles, as well as prevention, intervention, and improvement programmes in education centres where this kind of situations is identified.

Knowing not just these characteristics but also other socio-demographic variables, as well as personality traits that can predispose students to suffer abuse/abuse other schoolmates, would drive us forward to be ten steps ahead of bullying and allow us to be better prepared to fight against this phenomenon that has been overlooked for years.

The authors of this study are a group of researchers working on the university, and they have detected cases of bullying at that educational level. This problem has attracted a professional and personal interest in dealing with a public health problem. In addition to this, our region is the community with the largest number of complaints by bullying. Therefore, it is a duty as a researcher and professor to evaluate possible predictor tools to deal with this phenomenon, which affects the infantile and young population and its repercussion with serious sequels in adulthood.

A key weapon in the battle against bullying is school nurses, who have the skills and competences needed for prevention, because their closeness to students, from the point of view of health based on working with assets, puts them in a privileged position for early detection. Therefore, for the educational community to give more relevance to the implications for clinical practice of this and other research on the subject, it is necessary to introduce the figure of the school nurse globally.

## Figures and Tables

**Table 1:** Descriptive data on the phenomenon of bullying.

Category	Subcategories	% (*n*)
Student profile (*n* = 93)	Aggressor	14.0 (*n* = 13)
	Victim	14.0 (*n* = 13)
Type of abuse (*n* = 93)	No	0 (*n* = 0)
	Insulting/threatening	32.3 (*n* = 30)
	Physical abuse	10.8 (*n* = 10)
	Rejection	44.1 (*n* = 41)
	Other	12.9 (*n* = 12)
Place where abuse occurs (*n* = 93)	No	2.2 (*n* = 2)
	In the classroom	53.8 (*n* = 50)
	At the playground	20.4 (*n* = 19)
	In the corridors	6.5 (*n* = 6)
	Other places	17.2 (*n* = 16)
Frequency of abuse (*n* = 90)	Every day	8.9 (*n* = 8)
	Once/twice a week	22.2 (*n* = 20)
	Rarely	50.0 (*n* = 45)
	Never	18.9 (*n* = 17)
Severity (*n* = 87)	Hardy or non-severe	42.5 (*n* = 37)
	Somewhat severe	36.8 (*n* = 32)
	Quite severe	14.9 (*n* = 13)
	Very severe	5.7 (*n* = 5)
Safety in the education centre (*n* = 90)	Unsafe or hardly safe	6.7 (*n* = 6)
	Somewhat safe	20.0 (*n* = 18)
	Quite safe	33.3 (*n* = 30)
	Very safe	40.0 (*n* = 36)

*Note*: *n* = participant sample.

**Table 2:** Descriptive data on the personality variables according to EPQ-J questionnaire.

Dimension	Categories	% (*n*)
N	Highly stable (0–4 points)	8.6 (*n* = 8)
	Stable tendency (5–9 points)	32.3 (*n* = 30)
	Stable (10 points)	9.7 (*n* = 9)
	Unstable tendency (11–15 points)	32.3 (*n* = 30)
	Unstable (16–20 points)	17.2 (*n* = 16)
E	Highly stable (0–5 points)	0 (*n* = 0)
	Stable tendency (6–11 points)	8.6 (*n* = 8)
	Stable (12 points)	2.2 (*n* = 2)
	Unstable tendency (13–18 points)	31.2 (*n* = 29)
	Unstable (19–24 points)	58.1 (*n* = 54)
P	Highly stable (0–3 points)	74.2 (*n* = 69)
	Stable tendency (4–7 points)	21.5 (*n* = 20)
	Stable (8 points)	4.3 (*n* = 4)
	Unstable tendency (9–12 points)	0 (*n* = 0)
	Unstable (13–17 points)	0 (*n* = 0)
S	Sincerity (0–10 points)	44.1 (*n* = 41)
	Social dissimulation (11–20 points)	55.9 (*n* = 52)

*Note*: E = extraversion; N = neuroticism; *n* = participant sample; P = psychoticism; S = sincerity.

**Table 3:** Correlations of personality variables, according to the EPQ-J questionnaire, and variables related to the characteristics of the perception of abuse, according to the Bull-S test, with the characteristics of the phenomenon of bullying and the rest of variables used in the study.

	N	E	P	S	AB
Victim or aggressor profile	0.615 *	0.445	0.370	0.474 *	0.620 **
N	1	0.488	0.216	0.275	0.529 **
E	0.448	1	0.250	0.168	0.319
P	0.216	0.250	1	0.292 *	0.180
S	0.275	0.168	0.292*	1	0.243
AB	0.529 **	0.319	0.180	0.243	1
	**Type of Abuse**	**Place**	**Frequency**	**Severity**	**Safety**
Type of abuse	1	0.688 **	0.376 **	0.201	0.193
Place	0.688 **	1	0.665 **	0.587 **	0.409
Frequency	0.376 **	0.665 **	1	0.254 *	0.287 **
Severity	0.201	0.587 **	0.254 *	1	0.244
Safety	0.193	0.409	0.287 **	0.244	1

*Note*: AB = anti-social behaviour; E = extraversion; N = neuroticism; P = psychoticism; S = sincerity. * *p* < 0.05, ** *p* < 0.01.
